# Lower-Limb Passive Heat Maintenance Combined With Pre-cooling Improves Repeated Sprint Ability

**DOI:** 10.3389/fphys.2018.01064

**Published:** 2018-08-03

**Authors:** C. Martyn Beaven, Liam P. Kilduff, Christian J. Cook

**Affiliations:** ^1^Faculty of Health, Sport and Human Performance, University of Waikato, Hamilton, New Zealand; ^2^Applied Sports, Technology, Exercise and Medicine Research Centre, Swansea University, Swansea, United Kingdom; ^3^School of Sport, Health and Exercise Sciences, Bangor University, Bangor, United Kingdom; ^4^Hamlyn Centre, Institute of Global Health Innovation, Imperial College London, London, United Kingdom; ^5^University of Canberra Research Institute for Sport and Exercise, University of Canberra, Canberra, ACT, Australia

**Keywords:** warm-up, thermoregulation, ice slurry, survival garment, repeated sprint performance, fatigue, rugby sevens

## Abstract

Pre-conditioning strategies to potentiate performance are a common feature of pre-competition routines. The elevation of muscle temperature is seen as a vital component of preparing for physical performance, while pre-cooling strategies have been adopted to offset fatigue during repeated efforts. We investigated the individual and combined effects of a passive heat maintenance strategy and the ingestion of an ice-water slurry on repeated sprint performance. In a random cross-over design, 12 professional male athletes performed 5 × 40 m maximal running sprints under one of four conditions following a standardized warm-up: 15-min passive rest (Control); wearing a lower-body survival garment (HEAT); consuming a 500 mL ice slushy (COLD); or wearing the survival garment and consuming the slushy (H+C). Measures of sprint speed, fatigue, heart rate, and rectal temperature were collected. Compared to COLD: HEAT improved Sprint 1 (ES: 0.84; *p* = 0.05), but negatively impacted Sprint 4 (ES: -0.87; *p* = 0.08), and Sprint 5 (ES: -1.57; *p* = 0.002). H+C was faster than Control for every sprint (ES: 0.28 to 0.66), clearly faster than COLD on Sprints 1–3 (ES: 0.73 to 0.54), and clearly faster than HEAT on Sprints 4 and 5 (ES: 1.31 and 1.87). Fatigue was greatest after the HEAT intervention with a large correlation between fatigue and rectal temperature (*r* = 0.66; *p* = 0.0204). While there are undoubtedly peripheral effects of cooling and heating on various aspects of muscle function and fatigue, understanding the integration of psychophysiological homeostatic feedback loops relating to a combined warming and cooling intervention may benefit sports in which repeat sprints are performed.

## Introduction

Interventions to acutely enhance athletic performance have been termed pre-conditioning strategies (Kilduff et al., 2013a), and appropriately implemented warm-up combined with passive heat maintenance has been demonstrated to elicit positive performance outcomes (Kilduff et al., 2013b). The positive effects of appropriate warm-up, however, are scarcely a new phenomenon. Asmussen and Böje (1945) were able to demonstrate that the ability to perform work was associated with the temperature of the working muscle and similar effects were observed via both active (cycling) and passive (diathermy or water immersion) interventions. Similarly in a swimming study, Muido (1946) wrote that in terms of beneficial performance outcomes, he could record “no difference between the active warming of the organism by preliminary work and the passive warming of the organism." Interestingly though, the studies of Asmussen and Böje (1945) and Muido (1946) disagree on the mediator of the effects, with either elevated muscle temperature or core temperature being declared the essential determinant of beneficial performance outcomes.

It is noteworthy that the study by Muido (1946) also demonstrated a negative effect of cold water immersion (21°C) on swimming performance times (-3.6 to -6.3%). Some 30 years later, Bergh and Ekblom (1979) elegantly demonstrated that jump performance (4.2% ⋅°C^-1^) and peak cycling sprint speed (4.4% ⋅°C^-1^) performance was positively related to muscle temperature. In contrast, it is apparent that the endurance capacity of muscles is negatively impacted by elevated temperatures (Edwards et al., 1972), with clear detrimental effects on exercise performance as a result of high environmental temperatures (Hargreaves, 2008). Specifically, environmental heat stress is associated with a faster rate of fatigue (Sargeant, 1987; Ball et al., 1999), and impaired repeated sprint performance (Drust et al., 2005; Skein et al., 2012). To combat the negative effects of heat on intense exercise, reports have detailed pre-cooling strategies effective in enhancing exercise time-to-exhaustion (Brück and Olschewski, 1987), time-trial performance (Yeargin et al., 2006), and mean power output (Marsh and Sleivert, 1999). Pre-cooling strategies have also been shown to be effective at improving motor skill performance even in a thermally non-challenging environment (Desai and Bottoms, 2017). The studies of Marsh and Sleivert (1999) and Yeargin et al. (2006) were notable in that the pre-cooling intervention consisted of cold water immersion applied to the torso only in an effort to avoid any direct negative effects of cooling on muscle temperature. The ingestion of an ice slurry also avoids direct cooling on the musculature and has been demonstrated to have positive effects on exercise performance analogous to those seen with cold water immersion (Siegel et al., 2012).

Thus, it apparent that elevated muscle temperature (via active or passive heating and/or maintenance) can enhance explosive muscle actions but may also have negative implications for fatigue and repeated efforts. Conversely, pre-cooling strategies targeted to minimize increases in core body temperature have the potential to enhance fatigue resistance but also negatively impact upon the ability of the muscle to produce force rapidly. Herein, we tested the hypothesis that sprint performance is enhanced through an active warm-up with passive heat maintenance but at the cost of a greater degree of fatigue, while a pre-cooling ice slurry intervention would decrease sprint performance, but reduce fatigue in a repeated sprint protocol. It was further hypothesized that a combination of the warm-up and pre-cooling interventions would improve both early sprint performance and fatigue resistance compared to a control where no additional preconditioning strategies were performed after warm-up.

## Materials and Methods

### Subjects

Twelve professional male rugby sevens athletes, aged between 20 and 24 years (mean ± SD age: 21.5 ± 1.3 years, body mass: 96.2 ± 9.3 kg, height: 1.85 ± 0.04 m) were recruited to this study which was approved by the institutional Research Ethics Committee. All participants provided written informed consent prior to their participation in accordance with the Declaration of Helsinki.

### Design

The athletes visited a temperature controlled gymnasium (25°C and 60% humidity) where they were accustomed to training, on five occasions separated by a minimum of 3 days. Two control sessions and three experimental sessions were performed in a randomized, counter-balanced cross-over design. All sessions were performed at the same time of day avoid the confounding influence of circadian variability on the outcome measures.

### Methodology

**FIGURE 1:**
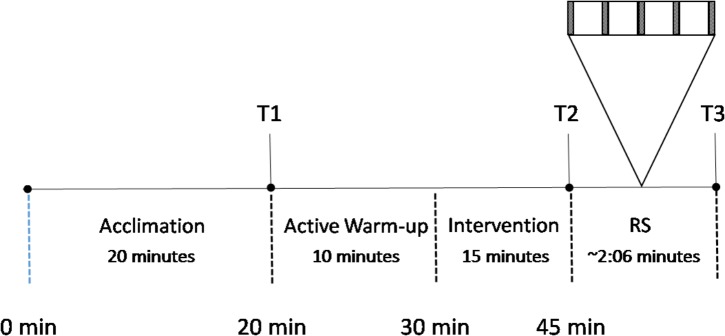
Experimental protocol. RS, repeated sprint exercise (5 × 40 m running sprint); T1, prior to warm-up; T2, prior to the RS; T3, post RS.

The control sessions consisted of arriving at the gymnasium where they were allowed 20 min to prepare for the session (e.g., changing into appropriate clothing). The athletes were then lead through a standardized active warm-up (10 min of increasing intensity running drills and short sprints) wearing team issue apparel (shorts and a lightweight shirt). The athletes then rested passively for 15 min before performing a repeated sprint protocol (RS) that they were previously familiarized with (5 × 40 m maximal running sprint efforts initiated every 30 s; see **Figure 1**). All sprints were performed on an indoor running track and time to complete each sprint was recorded by electronic timing lights (SmartSpeed Pro, Fusion Sport, Australia). The protocol during the three experimental trials was identical to the control sessions (CON) except that during the 15 min following the active warm-up, the athletes performed one of three interventions: wearing a lower-body heat reflective survival garment (Blizzard Survival Garments, United Kingdom, (Cook et al., 2012): HEAT); consuming a 500 mL flavored, non-calorific ice slushy (COLD); or wearing the reflective garment and consuming the ice slushy (H+C). Participants were allowed to self-select the rate of consumption of the ice slushy. Heart rate (Polar M400, Polar Electro Oy, Finland) and rectal temperature (YSI compatible thermistor inserted in the rectum to 10 cm) data were collected at three time points: prior to warm-up (T1), prior to the RS (T2), and upon completion of the RS (T3; **Figure 1**).

The athletes were requested to keep a food diary during the 24-h period prior to their first exercise trial and to replicate this intake prior to each subsequent trial. The athletes were also asked not to consume any food or beverages, other than water, during the 2-h period prior to testing. Additionally, participants were asked not to consume alcohol or to perform vigorous exercise in the 24-h prior to each session. Failure to comply resulted in rescheduling.

### Statistical Analysis

Requisite transformations of the physiological and performance data (log transformation) was performed prior to statistical analysis to reduce bias arising from non-uniformity of error (Hopkins et al., 2009). Separate analyses of variance procedures were performed to assess differences in physiological responses between the interventions. The magnitude of between-condition differences in the means were expressed as an effect size (ES), which were calculated using the pooled standard deviations. Threshold values for ES statistics were >0.2 (small), >0.6 (moderate), >1.2 (large), >2.0 (very large), and >4.0 (extremely large). Confidence intervals (90%) for the (true) mean changes or between-group differences were estimated (Hopkins et al., 2009). Quantitative chances of the likelihood of between condition differences were assessed qualitatively as follows: ≤1% almost certainly not, >1–5% very unlikely, >5–25% unlikely, >25–75% possible, >75–95% likely, >95–99 very likely, >99% almost certain. The effect was deemed ‘clear’ if its confidence interval did not overlap the thresholds for small positive and negative effects (Hopkins et al., 2009). Bi-variate relationships between variables of interest were examined via multiple regression to control for between subject variation (r). Magnitudes of correlations were interpreted using thresholds of 0.1, 0.3, 0.5, 0.7, and 0.9 for small-, moderate-, large-, very large-, and nearly perfect correlations, respectively (Hopkins et al., 2009). Significance was set at an alpha level of *p* ≤ 0.05. The calculated Typical Error for the repeated sprint protocol for the sprints was 0.05 s (range 0.04 to 0.08 s) and for the cumulative performance time was 0.14 s (0.8% as a CV).

## Results

**FIGURE 2:**
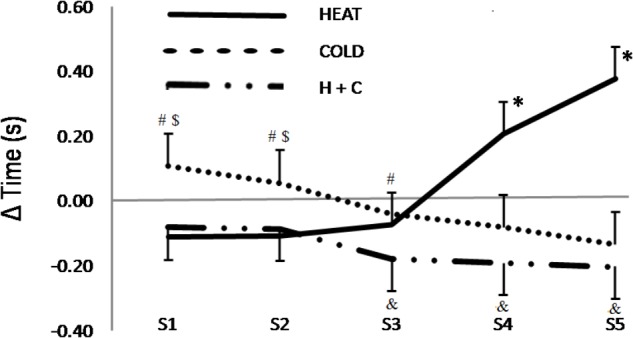
Relative performance - Sprint speeds relative to Control for Sprints 1 to 5. Control: 15-min passive rest prior to exercise; HEAT: utilized a lower-body reflective survival garment prior to exercise; COLD: consumed a 500 mL ice slushy prior to exercise; H+C: wore the reflective garment and consumed the ice slushy prior to exercise. ^∗^: substantially slower relative to Control; ^#^: COLD substantially slower than H+C; ^$^: COLD substantially slower than HEAT; ^&^: H+C substantially faster than Control.

All athletes completed each of the five sessions. There were no differences between the two control sessions at any time point for any of the outcome measures; therefore, the average of these data are presented for clarity. Sprint times are presented in **Table 1**. Compared to the CON, the HEAT intervention improved Sprint 1 (ES: -0.39), 2 (ES: -0.37), and 3 (ES: -0.25) (**Figure 2**); but clearly performed worse on Sprint 4 (ES: 0.68) and Sprint 5 (ES: 1.21, *p* = 0.0149). The COLD intervention slowed Sprint 1 (ES: 0.45) and 2 (ES: 0.26), saw trivial differences in Sprint 3 and 4, and was faster than CON in Sprint 5 (ES: -0.36). Compared to COLD, HEAT improved Sprint 1 (ES: 0.84; *p* = 0.05), but negatively impacted Sprint 4 (ES: -0.87; *p* = 0.08) and 5 (ES: -1.57; *p* = 0.002). H+C was faster than Control for every sprint (ES: 0.28 to 0.66), clearly faster than COLD on Sprints 1 to 3 (ES: 0.73 to 0.54), and clearly faster than HEAT on Sprints 4 and 5 (ES: 1.31 and 1.87, *p* = 0.0067 and 0.0001). The cumulative sprint times (**Table 1**) showed no substantial differences between the CON, COLD, and HEAT interventions; but H+C clearly improved overall performance compared to CON (ES: -0.42), COLD (ES: -0.43), and HEAT (ES: -0.59, *p* = 0.0825).

**Table 1:** Sprint times (s) for each sprint in the repeated-sprint protocol and cumulative time.

	Sprint 1	Sprint 2	Sprint 3	Sprint 4	Sprint 5	Cumulative
CON	5.31 (0.30)	5.38 (0.30)	5.53 (0.34)	5.63 (0.37)	5.73 (0.37)	27.58 (1.58)
HEAT	5.20 (0.28)	5.28 (0.28)	5.46 (0.29)	5.84 (0.39)	6.10 (0.35)	27.87 (1.52)
COLD	5.43 (0.31)	5.46 (0.32)	5.52 (0.35)	5.57 (0.34)	5.62 (0.35)	27.60 (1.66)
H+C	5.23 (0.28)	5.30 (0.28)	5.36 (0.27)	5.44 (0.27)	5.53 (0.28)	26.85 (1.37)

*Data are presented as mean (standard deviation).*

**FIGURE 3:**
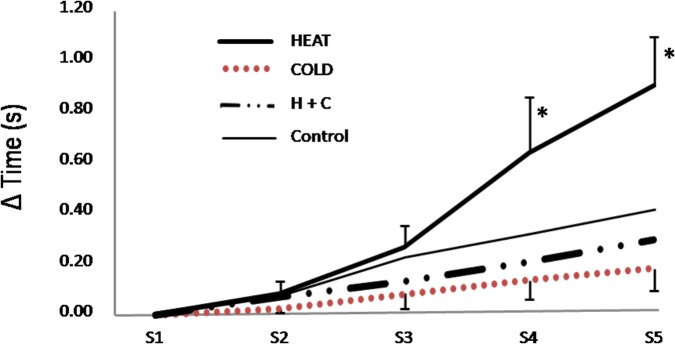
Fatigue profile - Sprint speeds relative to Sprint 1 for Sprints 1 to 5. Control: 15-min passive rest prior to exercise; HEAT: utilized a lower-body reflective survival garment prior to exercise; COLD: consumed a 500 mL ice slushy prior to exercise; H+C: wore the reflective garment and consumed the ice slushy prior to exercise. ^∗^: substantially more fatigue relative to all other interventions.

The rate of fatigue, as calculated from time difference between Sprint 5 and Sprint 1, was substantially lower following the COLD intervention compared to the H+C (1.4%; ES: 0.24 ± 0.18; *p* = 0.0293), CON (3.2%; ES: 0.58 ± 0.27; *p* = 0.0015), and HEAT (8.8%; ES: 1.57; *p* = 1.22 × 10^-6^; see **Figure 3**). Specifically, the fatigue was greatest after the HEAT intervention at every time point with moderate to large differences observed during Sprint 4 (ES: 1.04 to 1.37) and Sprint 5 (ES: 1.57 to 2.00; see **Figure 3**). There was a large correlation between RS fatigue and rectal temperature at T2 (*r* = 0.66; *p* = 0.0204).

**FIGURE 4:**
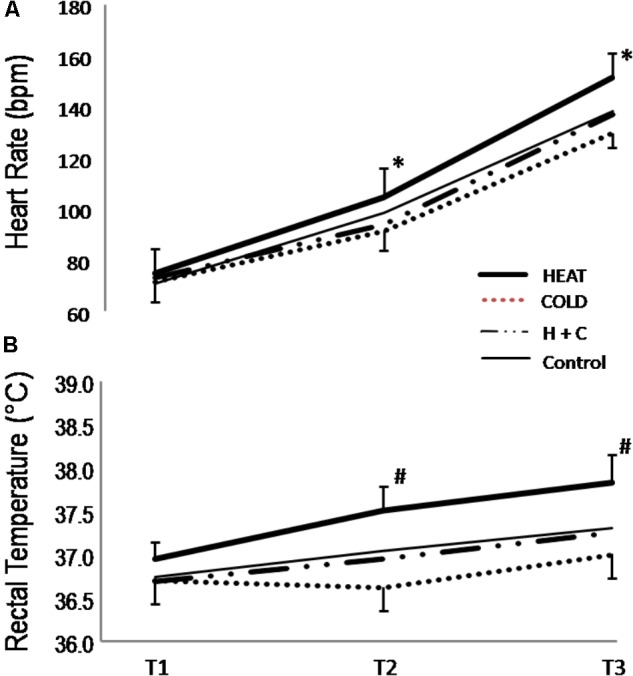
Physiological responses – Heart rate and rectal temperature. **(A)** Heart rate and **(B)** rectal temperature. Control: 15-min passive rest prior to exercise; HEAT: utilized a lower-body reflective survival garment prior to exercise; COLD: consumed a 500 mL ice slushy prior to exercise; H+C: wore the reflective garment and consumed the ice slushy prior to exercise. ^∗^: substantial difference between COLD and both Control and HEAT interventions. ^#^: substantial difference between HEAT and all other interventions.

Physiological outcome measures are presented in **Figure 4**. Heart rate was increased from T1 to T3 as expected with the highest heart rate following the HEAT intervention (151 ± 11 bpm) and the lowest following the COLD (129 ± 9 bpm; see **Figure 4A**). There was a substantial difference at T2 between the COLD and both the CON (7.5 ± 5.9%; ES: 0.92 ± 0.68; *p* = 0.0302) and the HEAT (14.1 ± 7.1%; ES: 1.30; *p* = 0.0036) interventions; and at T3 these differences increased (CON: ES: 1.09; *p* = 0.0116; and HEAT: ES: 2.19; *p* = 1.59 × 10^-5^). Rectal temperature following the HEAT intervention was higher at T2 than CON (1.2 ± 0.5%; ES: 1.58; *p* = 0.0006), COLD (2.4 ± 0.5%; ES: 3.11; *p* = 1.03 × 10^-7^), and H+C (1.5 ± 0.5%; ES: 2.13; *p* = 2.25 × 10^-5^; see **Figure 4B**); and these differences persisted after the RS (CON: ES: 1.49, *p* = 0.0012; COLD: ES: 2.42, *p* = 6.35 × 10^-6^; H+C: ES: 1.71, *p* = 0.0004).

## Discussion

The combination intervention of the ingestion of 500 mL of an ice slurry with passive heat maintenance using a reflective survival garment effectively enhanced both initial sprint speed and fatigue resistance in our repeated sprint protocol performed in a heated environment (25°C and 60% relative humidity). As a result, the cumulative time to complete the task was superior in this combination intervention than when either of the interventions were performed in isolation. Specifically, the HEAT intervention enhanced initial sprint performance but exhibited a greater performance decrement, while the COLD intervention negatively impacted upon initial sprint performance but imparted a degree of fatigue resistance.

It is well established that both passive and active elevation of muscle temperature can enhance the capacity of the muscle to perform work (Asmussen and Böje, 1945; Muido, 1946; Sargeant, 1987; Ball et al., 1999; Linnane et al., 2004; Gray et al., 2006; Skein et al., 2012; West et al., 2013). Further, both passive (Cook et al., 2012; Russell et al., 2015) and active (Mohr et al., 2004) heat maintenance strategies have demonstrated meaningful enhancements in sprint performance in athletes. Proposed mechanisms for the performance enhancements are an elevated rate of adenosine triphosphate (ATP) turnover and muscle fiber conduction velocity in type IIa muscle fibers (Gray et al., 2006), a rightward shift in the power-velocity relationship in type I muscle fibers (Ball et al., 1999; Linnane et al., 2004) and beneficial effects on contractility (Davies and Young, 1983; Morrison et al., 2004), viscosity (Asmussen and Böje, 1945), and the rate of enzymatic and metabolic processes (Asmussen and Böje, 1945; Edwards et al., 1972).

While no neuromuscular or metabolic data were included in the current study, from a performance perspective passive heat maintenance following an active warm-up improved initial sprint performance (**Figure 2**). Conversely, the elevated rectal temperature seen in HEAT significantly and negatively affected repeated running performance with the highest fatigue index and a positive relationship between performance decrement and rectal temperature. Given the proposed mechanisms above, it is apparent that an increase in ATP depletion and a shift toward a metabolic profile consistent with less fatigue-resistant muscle fiber type has the potential to impact repeated sprint performance.

An objectively similar degree of fatigue to that observed in the HEAT intervention has previously been reported when a series of sprints were performed in a hot environment (Drust et al., 2005). These authors concluded that the simultaneous elevation core and muscle temperature negated any beneficial effect of elevated muscle temperature on the ability to perform intense repeated sprint exercise. The 0.87 ± 0.43°C (range 0.0 to 1.5°C) elevation in rectal temperature observed in the HEAT condition (**Figure 4B**) in the current study is representative of an elevated core temperature that has the potential to further impact exercise capacity by diminishing the drive to exercise (Brück and Olschewski, 1987; Ückert and Joch, 2007). Of note, Todd et al. (2005) demonstrated that passive hyperthermia (38.5°C) induced a significant decrease in voluntary force which they attributed to a failure of voluntary drive from the motor cortex. While we accept that 25°C and 60% relative humidity may not be considered extreme environmental conditions, we did observe rectal temperatures ≥38.0°C in six out of twelve of the participants following exercise in the HEAT intervention. In addition, the high relative exercise intensity (Sawka et al., 2010) and reduced thermoregulatory efficiency (impeded heat dissipation) associated with intermittent exercise performed above VO_2MAX_ (Ekblom et al., 1971) likely combined to present a thermoregulatory challenge.

To counteract these negative impacts of hyperthermia, researchers have implemented pre-cooling strategies to confer thermoregulatory and performance benefits (Castle et al., 2006; Yeargin et al., 2006; Ückert and Joch, 2007). However, cooled muscle is associated with decreased voluntary power output (Davies and Young, 1983) and contractile slowing (Bigland-Ritchie et al., 1992). Skein et al. (2012) highlighted the opposing effects of a pre-cooling strategy (ice bath immersion) when they reported slowed initial sprint times but an improved maintenance of voluntary torque and distance covered when compared to a passive heat strategy. Although the magnitude of the effect of pre-cooling was less than that observed by Skein et al. (2012), our data demonstrates that the COLD intervention negatively affected early sprint performance, despite our orally delivered pre-cooling strategy avoiding any potential for direct cooling of the muscle to impair muscle performance. The use of an ice slurry ingestion, however, retains the potential to increase blood flow to the active muscles (Marsh and Sleivert, 1999), core temperature afferent signaling to the brain, and act as a heat sink due to the enthalpy of fusion (Siegel et al., 2010). Thus, our ice slurry intervention data are consistent with a both positive (fatigue resistance) and negative (slowed initial sprint) perceptual effects.

From the work of Castle et al. (2006), it can be inferred that the degree of afferent feedback is an important consideration with respect to the efficacy of a pre-cooling strategy, with the vest intervention that provided the least sensory information eliciting little benefit in peak power output. While the COLD intervention was effective in eliciting a decrease in rectal temperature (**Figure 4B**), the H+C and CON interventions were indistinguishable at all measurement points despite the observed performance benefits of the H+C intervention. Thus, the ingestion of an ice slushy, with no potential for a direct impact on muscle temperature but a substantial degree of sensory input, was effective in altering the performance outcome variables (**Figures 2**, **3**). It is important to note that a pre-cooling protocol using an ice pack applied to the neck, has been shown to be effective in improving table tennis skill performance by lowering thermal sensation in a thermally non-challenging environment (21.3°C and 44.5% relative humidity) (Desai and Bottoms, 2017). Further, it is worth noting that the perception of cooling in the oral cavity associated with a 10 s menthol swill (without ingestion) has been shown to be an effective in “altering psychophysical processes” and improving exercise capacity (Mündel and Jones, 2010).

While we acknowledge the limitations of the current research in that brain, muscle, and skin temperatures were not assessed, herein we describe a practical and effective strategy to enhance repeated sprint performance. Clearly, invasive measures of muscle temperature would aid in interpreting the current data by giving an indication of the localized changes occurring at the muscle. We also note here that there is the potential for the ice slushy intervention to have provided a positive effect due to hydration, over-and-above its cooling effects and that this COLD intervention could elicit a positive psychological effect. Understanding the underlying physiology and psychophysiology of combined warming-cooling approaches may benefit sports in which repeat efforts are performed under heat loads commonly experienced in sports such as rugby sevens, hockey, and football. The ability to have specific impacts via preconditioning on speed and fatigue profiles has important ramifications for a range of sports where high intensity efforts are interspersed with relatively low intensity periods.

## Perspective

From a performance and practicality standpoint, the combination of both ice slurry ingestion and localized muscle temperature maintenance enhanced initial sprint speed (**Figure 2**), overall work capacity, and fatigue resistance (**Figure 3**). The current work builds upon previous research by Sleivert et al. (2001), who attempted to elucidate the antagonistic effects of muscle temperature-based pre-conditioning and pre-cooling, and concluded that muscle temperature directly impacted the contractile function of the muscle. It is interesting to reflect on the contrast between the conclusions of Nybo and Nielsen (2001) and Sleivert et al. (2001), which mirror the early contrasting conclusions of Asmussen and Bøje and Muido. Further, work from a group including Sleivert (Thomas et al., 2006), concluded that impaired neuromuscular activation occurred independently of changes in muscle temperature, and instead resulted from a “central failure.” It is apparent that fatigue is a complex teleological phenomenon integrating psychophysiological inputs culminating in the “conscious awareness of changes in subconscious homeostatic control systems” (St Clair Gibson et al., 2003). While there are undoubtedly peripheral effects of cooling and heating on various aspects of muscle function and fatigue, the integration of a range of signals determines the overall commitment of resources based on psychophysiological homeostatic feedback loops, teleoanticipation, and preconceived expectations.

## Author Contributions

CB, LK, and CC contributed substantially to the conception and design of the work; the acquisition, analysis, and interpretation of data for the work; drafted the work and revised it critically for important intellectual content; approved the final version to be published; and agreed to be accountable for all aspects of the work in ensuring that questions related to the accuracy or integrity of any part of the work are appropriately investigated and resolved.

## Conflict of Interest Statement

The authors declare that the research was conducted in the absence of any commercial or financial relationships that could be construed as a potential conflict of interest.
